# An archaeal family-B DNA polymerase variant able to replicate past DNA damage: occurrence of replicative and translesion synthesis polymerases within the B family

**DOI:** 10.1093/nar/gku683

**Published:** 2014-07-24

**Authors:** Stanislaw K. Jozwiakowski, Brian J. Keith, Louise Gilroy, Aidan J. Doherty, Bernard A. Connolly

**Affiliations:** 1 Genome Damage and Stability Centre, School of Life Sciences, University of Sussex, Brighton BN1 9RQ, UK; 2 Institute of Cell and Molecular Biosciences, University of Newcastle, Newcastle upon Tyne, NE2 4HH, UK

## Abstract

A mutant of the high fidelity family-B DNA polymerase from the archaeon *Thermococcus gorgonarius* (Tgo-Pol), able to replicate past DNA lesions, is described. Gain of function requires replacement of the three amino acid loop region in the fingers domain of Tgo-Pol with a longer version, found naturally in eukaryotic Pol ζ (a family-B translesion synthesis polymerase). Inactivation of the 3′–5′ proof-reading exonuclease activity is also necessary. The resulting Tgo-Pol Z1 variant is proficient at initiating replication from base mismatches and can read through damaged bases, such as abasic sites and thymine photo-dimers. Tgo-Pol Z1 is also proficient at extending from primers that terminate opposite aberrant bases. The fidelity of Tgo-Pol Z1 is reduced, with a marked tendency to make changes at G:C base pairs. Together, these results suggest that the loop region of the fingers domain may play a critical role in determining whether a family-B enzyme falls into the accurate genome-replicating category or is an error-prone translesion synthesis polymerase. Tgo-Pol Z1 may also be useful for amplification of damaged DNA.

## INTRODUCTION

DNA polymerases are core components of multi-protein assemblies responsible for all aspects of cellular DNA metabolism including replication, recombination, translesion synthesis and repair (1–10). Comparison of amino acid sequences, in combination with structural information, allow assignment of the enzymes into seven families: A, B, C, D, X, Y and RT (reverse transcriptase) (1–4). However, based on biological function, a distinction may be drawn between replicative (mainly found in families A, B, C and D) and translesion synthesis (usually confined to families X and Y) polymerases. Replicative DNA polymerases exhibit a ‘tight fit’ for their DNA and dNTP substrates and are superbly adapted to form correct Watson–Crick base pairs, resulting in pronounced fidelity. The strong preference of these enzymes to produce A/T or G/C base pairs leads to marked inhibition by abnormal bases present at sites of DNA damage, features which usually cause replicative polymerases to stall (8–11). In contrast, translesion and repair polymerases possess ‘looser’ solvent exposed active sites, which tolerate aberrant DNA features much better. A large number of translesion DNA polymerases are known for their common features of having a preference to read through a particular damaged base/structure (8–11). The more open nature of these enzymes leads to lowered accuracy and polymerisation is often largely distributive, i.e. only a few bases are added per binding event. Such specialised features are an obvious disadvantage during replication of genomic DNA, but are beneficial when DNA translesion synthesis is required.

The B family of polymerases is the most abundant, with members in viruses, bacteria, archaea and eukaryotes (1,6–7). These polymerases are generally dedicated to genome replication as exemplified in the eukaryotes by three enzymes; Pol α, which initiates DNA synthesis (5) and Pols δ and ϵ, responsible for replication of lagging and leading strands, respectively (12,13). Structures are available for all three proteins and have revealed the presence of an active site superbly shaped for producing Watson–Crick base pairs which, when coupled with the proof-reading exonuclease activity, leads to faithful DNA synthesis (14–16). All archaea contain a family-B polymerase and in one kingdom, the crenarchaeota, it appears to be the only enzyme with properties compatible with DNA replication (7,17–18). Other archaeal kingdoms, e.g. the euryarchaeota, contain an unusual family-D polymerase (7,19–20) in addition to the family-B enzyme. Recent genetic experiments have shown that the family-B polymerase can be deleted with minimal changes to phenotype, whereas the family-D polymerase is essential (21,22) and so Pol D rather than Pol B may be used for replication in these species. However, the role each polymerase plays in the archaeal domain awaits determination with the same rigour as established for bacteria and eukaryotes. Regardless of the functions family-B polymerases serve in the archaea, they generally show high accuracy, much prized in polymerase chain reaction (PCR) applications (23–25). Although polymerases committed to handling damaged bases are mostly found in the X and Y families, the B-group contains two members dedicated to repair functions. Several, but not all, bacteria possess a family-B polymerase, the most thoroughly characterised being *Escherichia coli* DNA polymerase II (Eco-Pol II). This enzyme appears to be important in the re-start of replication forks that have been stalled by the encounter of blockages in the genome (26). Structural data show this polymerase has small cavities outside the active site, which are used to loop out damaged bases in the template strand, leading to better tolerance of their presence (27). Eukaryotes also contain Pol ζ, a unique family-B polymerase that plays a key role in the replication of damaged bases (28–30); a specialised repair polymerase first inserts a standard base opposite the lesion and Pol ζ is then able to extend the resulting mismatch (31). As expected, Pol ζ has much lower fidelity than Pols ϵ and δ (11).

**Figure 1.**
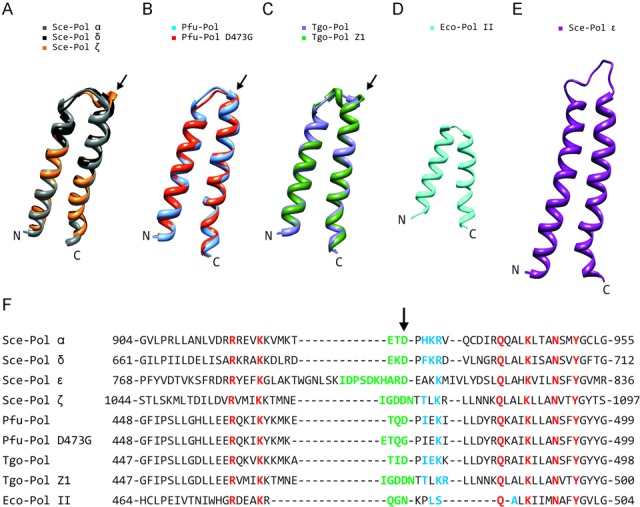
Comparison of fingers domains from family-B DNA polymerases. (**A**)–(**C**) Structural superimposition of the fingers regions from: (**A**) *Saccharomyces cerevisiae* Sce-Pol α (gray: pdb, 4FVM), Sce-Pol δ (black: pdb, 3IAY) and Sce-Pol ζ (orange: ‘Phyre2’ model); (**B**) *Pyrococcus furiosus* Pfu-Pol (blue: pdb, 2JGU) and Pfu-Pol (D473G) (red: ‘Phyre2’ model); (**C**) *Thermococcus gorgonarius* Tgo-Pol (purple: pdb, 1Tgo) and Tgo-Pol Z1 (green: ‘Phyre2’ model). With Sce-Pols α and δ, this region is virtually superimposable but the model of Sce-Pol ζ clearly shows an extended loop (arrowed). Similarly, the models of Pfu-Pol D473G and Tgo-Pol Z1 also indicate a slightly longer loop (arrowed) as compared to the wild types. (**D**) Fingers region of Eco-Pol II (cyan: pdb, 3K5O). (**E**) Fingers region of Sce-Pol ϵ (purple: pdb, 4M8O). (**F**) Amino acid alignments of the fingers domains. Amino acids in the loop region, based on the above structures/modelling, are coloured green. The arrow indicates the critical aspartic acid, which hydrogen bonds with amino acids (shown in blue) in the subsequent α-helix. The residues shown in red are very highly conserved and interact with incoming dNTPs. Sce: *Saccharomyces cerevisiae*; Pfu: *Pyrococcus furiosus*; Tgo: *Thermococcus gorgonarius*; Eco: *Escherichia coli*.

*Pyrococcus furiosus* (Pfu), and the closely related *Thermococcus gorgonarius* (Tgo), are hyperthermophiles from the euryarchaeal order *Thermococcales* (32), which furnishes most of the polymerases used in PCR applications that require high accuracy (23–25). Mutations to an aspartic acid, located in a three amino acid loop linking the two long α-helices that make up the fingers domain (Figure 1), decrease the accuracy of *Pyrococcus furiosus* Pol B (Pfu-Pol) (33). In both Pfu-Pol and Tgo-Pol, as previously documented (33), the critical aspartic acid makes a network of hydrogen bonds to a set of amino acids located in the following helix (Figure 1, Table 1). No structure exists for the most thoroughly investigated mutant, Pfu-Pol D473G (33), but a ‘Phyre2’ threading model suggests a loop extended by one amino acid with retention of only a single hydrogen bond (Figure 1, Table 1). The aspartic acid uses both side chain and peptide bond atoms for hydrogen bonding, explaining the persistence of an interaction with D473G. The accurate eukaryotic Pols α and δ have an almost identical fingers domain to the archaeal polymerases; amino acid sequence and structural comparisons both show a near superimposable three amino acid loop with retention of the aspartic acid and similar hydrogen bonding to downstream amino acids (Figure 1, Table 1). Sequence alignments suggest that Pol ζ has a longer loop and, although structural information is lacking, modelling is also consistent with elongation (Figure 1, Table 1). Two hydrogen bonds result, between D1070 (the D473 equivalent) and amino acids +3 (T1073) and +5 (K1075) along. A further manipulation to the fingers loop, commencing with a proof-reading exonuclease variant of Tgo-Pol, replaced the natural three amino acids with the longer sequence found in Sce-Pol ζ (Figure 1, Table 1). The resulting derivative, referred to as Tgo-Pol Z1, possessed reverse transcriptase activity and had low fidelity (34). It is probable that Tgo-Pol Z1 has an expanded flexible active site able to accommodate the non-B DNA structures of DNA–RNA heteroduplexes (35) formed when RNA is copied with dNTPs. Again, no structures have been determined for Tgo-Pol Z1 but a model is consistent with that of Sce-Pol ζ, i.e. a long loop with hydrogen bonds between the aspartate and succeeding residues (Figure 1, Table 1). The sequences and structures of the fingers domains of *Saccharomyces cerevisiae* polymerase epsilon (Sce-Pol ϵ) and bacterial Eco-Pol II are also shown in Figure 1. Although a Sce-Pol ϵ replicase possesses an extended loop and demonstrates only a single hydrogen bond between D807 (equivalent to D473) and downstream amino acids (Table 1). In contrast, although Eco-Pol II belongs in the translesion category, the fingers loop is short (three amino acids) and N485 (the spatial counterpart of D473) makes three hydrogen bonds with the succeeding α-helix. Some observations in Figure 1 and Table 1 are based on modelled rather than real structures and merit a degree of caution.

**Table 1.** Hydrogen bonds formed between the fingers domain loop aspartic acid or its equivalent (the amino acid shown in green and arrowed in Figure 1) and amino acids (shown in blue) in the subsequent α-helix

DNA polymerase	Data source	Number of H bonds	Hydrogen bonding partners^a^
Sce-Pol α	pdb, 4FVM	3	D929-H931
			D929-K932
			D929-R933
Sce-Pol δ	pdb, 3IAY	3	D686-F688
			D686-K689
			D686-R690
Sce-Pol ϵ^b^	pdb, 4M8O	1	D807-K811
Sce-Pol ζ^b^	‘Phyre2’ model	2	D1070-T1073
			D1070-K1075
Pfu-Pol	pdb, 2JGU	2	D473-I475
			D473-K477
Pfu-Pol D473G^b^	‘Phyre2’ model	1	G473-K477
Tgo-Pol	pdb, 1TGO	3	D472-I474
			D472-E475
			D472-K476
Tgo-Pol Z1^b^	‘Phyre2’ model	3	D473-T476
			D473-K478
			D473-R479
Eco-Pol II^c^	pdb, 3K5O	3	N485-L488
			N485-S489
			N485-A491

^a^The first residue shown is the aspartic acid (asparagine in the case of Eco-Pol II) in the loop region, the second the amino acids in the following α-helix. The underlined hydrogen bonds were found with a distance constraint <0.4 Å and an angle constraint <20°. The other hydrogen bonds had more relaxed constraints of <1 Å and <30°.

^b^These DNA polymerases possess a fingers loop that is longer than three amino acids.

^c^With Eco-Pol II the third residue in the loop is asparagine rather than aspartic acid.

Thus, experimental evidence suggests that perturbations to the fingers domain loop of high fidelity archaeal DNA polymerases results in features, including low fidelity and the ability to handle perturbed DNA structures, characteristic of translesion synthesis enzymes (33,34). However, there appears to be no straightforward correlation between loop length/loop-helix hydrogen bonds and the position of a polymerase within the replicative or translesion class (Figure 1, Table 1). Moreover the fidelity and translesion DNA synthesis ability of Tgo-Pol Z1 variant was not studied in detail before. Therefore, this publication thoroughly characterises Tgo-Pol Z1 in order to further elucidate any role of the loop region in determining replicative/translesion properties. In particular, the ability of this enzyme to insert bases opposite template strand lesions, and to further extend the resulting mismatches, has been determined. It is shown that introducing the Sce-Pol ζ loop into archaeal polymerases confers a strong ability to read through many damaged bases. This conversion of a high fidelity enzyme to a translesion polymerase sheds light on how polymerases within the single family-B may belong to either class and suggests a method for rational engineering of archaeal polymerases for amplification of partially degraded DNA.

## MATERIALS AND METHODS

### DNA polymerases

The preparation of Tgo-Pol Z1 has previously been described (34). This variant contains the Pol ζ fingers loop (Figure 1) and has the 3′–5′ proof-reading exonuclease activity disabled (exo^−^) by the mutation of D215A. For comparative purposes, the parental Tgo-Pol exo^−^ (D215A) was used. Throughout the main text of this publication the designations Tgo-Pol Z1 and Tgo-Pol are used, with the exo^−^ status of both not explicitly mentioned to prevent constant repetition. An exception is made in Table 2 as the full wild type Tgo-Pol (exo^+^) is also mentioned here. KlenTaq DNA polymerase was purchased from Bioline. *E. coli* DNA polymerase II (Eco-Pol II) (both exo^+^ and exo^−^) was a kind gift of Dr. Wei Yang (NIH, Bethesda, USA).

### DNA substrates

The oligodeoxynucleotides (supplied HPLC purified and desalted) used to prepare primer-templates were purchased from ATDBio (Southampton, UK). The modified bases incorporated into the template were: dSpacer (stable abasic site), (Glen Research 10-1913), *cis*-5R,6S thymidine glycol (Glen Research 10-1096) and *cis*-syn thymine cyclobutane dimer (Glen Research 11-1330). The sequence containing the thymine dimer (6–4) photoproduct was kindly provided by Professor Shigenori Iwai (Osaka University, Japan). All primers were labelled with hexachlorofluorescein at their 5′ ends to allow visualisation following gel electrophoresis. Primer-templates were annealed by heating equimolar amounts in 10 mM Tris, pH 7.5, 50 mM NaCl, 0.5 mM EDTA at 95°C for 5 min and then cooling slowly to room temperature.

**Figure 2.**
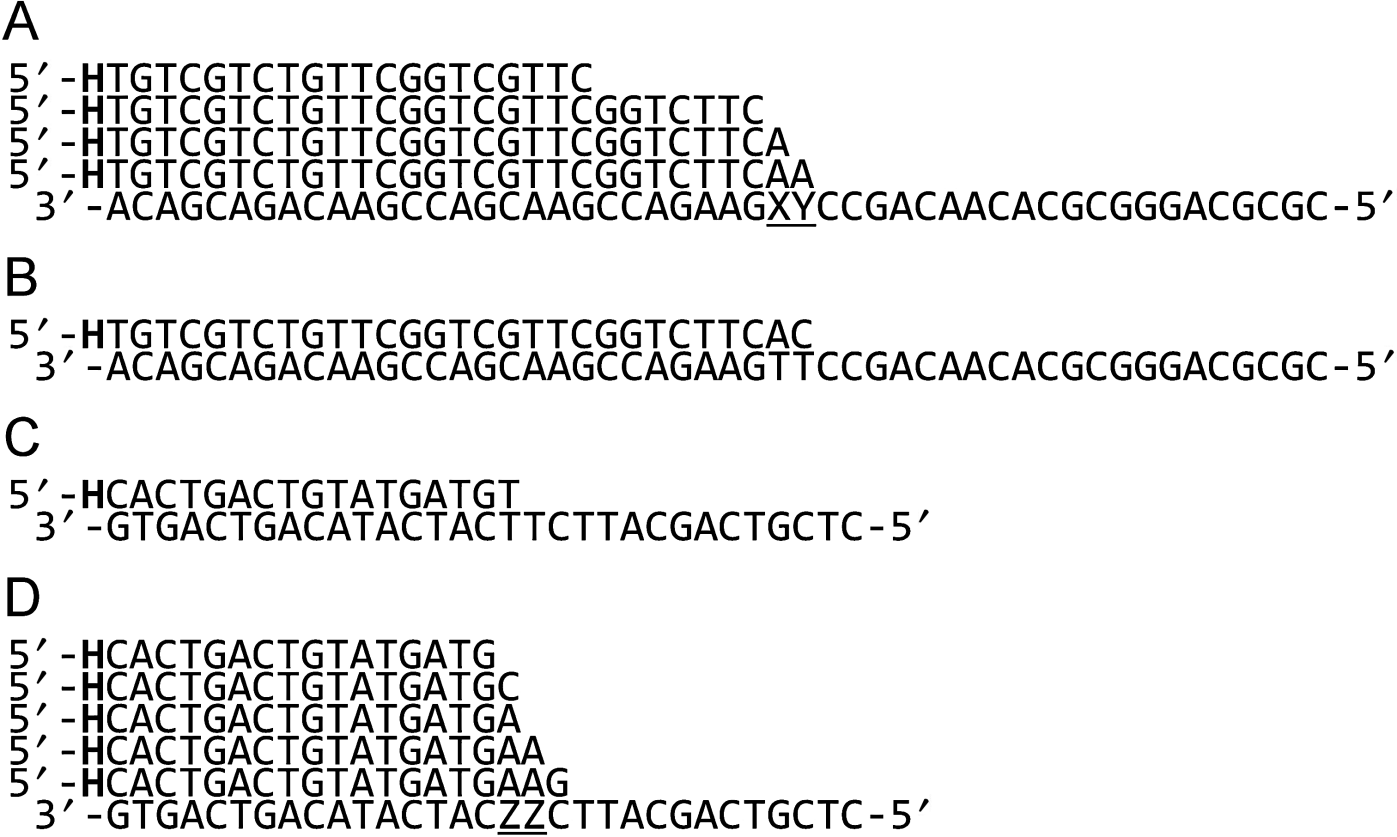
Oligodeoxynucleotides used in this study. (**A**) The 50 base template (written in 3′→5′ direction) containing various damaged bases (X = T, Y = T (control); X = T, Y = abasic site; X = T, Y = thymine glycol; X/Y = cis/syn thymine–thymine cyclobutane dimer) was hybridised with one of four primers (written in the 5′→3′ direction). The shortest primer allows running start read-through of the lesions. The longer primers allow extension to begin: (1) at the base immediately preceding the lesion; (2) directly opposite the abasic site or thymine glycol; (3) from either of the damaged bases in the thymine dimer. (**B**) and (**C**) Primer-templates designed to initiate DNA synthesis at mismatched C:T and T:T base pairs, respectively. (**D**) The substrates used to investigate the thymine 6–4 photoproduct dimer. The template strand (written in the 3′→5′ direction, ZZ represents the 6–4 photoproduct) was hybridised with one of four primers (written in the 5′→3′ direction). The primers allow synthesis to commence from: (1) the base directly before the lesion; (2) the 3′ base of the 6–4 photoproduct; (3) the 5′ base of the 6–4 photoproduct; (4) the base immediately following the lesion. All the primers shown in the figure were 5′-end labelled with hexachlorofluorescein (H).

The primer-templates used in these studies are illustrated in Figure 2. Most are based on templates 50 bases in length that contain a damaged base (abasic site, thymine glycol or *cis*-syn thymine–thymine cyclobutane pyrimidine dimer) 22 bases from the 5′ end (22 and 23 in case of the dimer). A number of primers, all labelled at their 5′-ends with hexachlorofluorescein for detection purposes, were applied. These could position the 3′ base used to initiate extension far upstream of the template modification (allowing running start read-through) or immediately in front of the lesion (allowing single base addition opposite the damaged base). Alternatively, the primer could terminate flush with the lesion, permitting study of extension from the resulting canonical base: damaged base mismatches (Figure 2A). Two primer-templates lack modified bases, designed, instead, to allow polymerisation to begin from a C:T or a T:T mismatch at 3′ end of the primer (Figure 2B and C). Due to the difficulties with synthesizing the thymine 6–4 photoproduct dimer, this lesion was present in a 30 base template, 13 and 14 bases from the 5′ end. Several primers that terminated just prior to the lesion, at the 3′ and 5′ bases of the lesion and immediately post lesion, were employed (Figure 2D).

### Polymerase activity assay

The PCR was employed to confirm thermostability and compare relative DNA polymerase activities of 3′–5′ exonuclease deficient (D215A) Tgo-Pol exo^−^ and its derivative Tgo-Pol Z1. Two different targets (∼0.4 kb and ∼2.5 kb) within the *Archaeoglobus fulgidus* genome were selected for amplification. The PCR reactions were carried out in 50 μl total reaction volume containing 20 mM Tris-HCl pH 8.8, 10 mM (NH_4_)_2_SO_4_, 50 mM KCl, 0.1% Triton X-100 (Sigma), 0.1 mg/ml bovine serum albumin (New England Biolabs), 2 mM MgCl_2_, 200 μM of all four dNTPs (Roche), 0.1 μM each of the forward and reverse primers (Eurofins MWG Operon, Germany), 100 ng *Archaeoglobus fulgidus* genomic DNA (DSM 4304, DMSZ, Germany) and 10 nM DNA polymerase. The primers (Eurofins MWG Operon, Germany) used were ATGGATGCAACTCTTGACAGGTTCTTC (forward primer) and AATGCCCTCAATTGGTGCAGCCACAAC (reverse primer, 0.4 kb amplicon)/CTTAACCAGCAAATCGTCTATGAAG (reverse primer, 2.5 kb amplicon). Amplification of the 0.4 kb target was achieved in 30 PCR cycles (30 s at 95°C, 35 s at 55°C and 30 s at 70°C). To amplify the 2.5 kb target, 30 PCR cycles (30 s at 95°C, 35 s at 55°C and 2.5 min at 70°C) were used. Additionally, KlenTaq DNA polymerase was used as a control. The PCR amplification was performed as described above with the following changes to the buffer: 10 mM Tris-HCl pH 8.3, 50 mM KCl, 1.5 mM MgCl_2_ and 25 nM KlenTaq polymerase. Amplicons were analysed on a 1% agarose gel with ethidium bromide staining.

### Primer extension assays

Extensions were performed in 20 μl volume containing 20 mM Tris, pH 8.8, 10 mM KCl, 10 mM (NH_4_)_2_SO_2_, 2 mM MgSO_4_, 20 nM primer-template and 10 nM DNA polymerase. For running start extensions, 50 μM of each of the four dNTPs (Roche) was used. In cases of single dNTP addition, 50 μM of the particular dNTP (Roche) under investigation was added. Reactions were carried out at 50°C for the times indicated in the Results section and then quenched by addition of an equal volume of 95% formamide/5% water containing 20 mM EDTA. Elongated products were resolved on 15% denaturing polyacrylamide gels containing 7 M urea. The resulting gels were scanned using a Fuji FLA-150 (Fuji, UK) operated in fluorescence mode.

### Plasmid-based *lacZα* fidelity assay

The fidelities of Tgo-Pol and Tgo-Pol Z1 were determined using pSJ2 in a plasmid-based *lacZα* reporter gene assay (36). The assay was carried out in a volume of 20 μl using the buffer given above for gel extensions with 40 fmol of gapped pSJ2, 250 μM of each dNTP and 100 nM of the polymerase. Polymerase-catalysed gap filling of pSJ2 was carried out at 70°C for 30 min and completion verified using an analytical digestion with EcoRI (Fermentas) restriction endonuclease and 1% agarose electrophoresis. With *E*. *coli* Pol II, pSJ3 (40 fmol) was used for fidelity determination. Reactions were performed in 20 μl of 40 mM Tris-HCl, pH 9.0, 50 mM KCl, 10 mM MgCl_2_, 1 mM DTT, 1 mM dNTPs and 100 nM enzyme for 1 h at 35°C. Fuller details have been reported as have the protocols for transformation of *E. coli* with the gap-filled plasmid and downstream blue/white analysis (36).

### Mutation spectrum of Tgo-Pol Z1

To determine the mutations introduced into amplified DNA by Tgo-Pol Z1 during the PCR, use was made of pSJ1, a plasmid that contains the *lacZα* indicator gene (37). Error prone amplification of the *lacZα* coding sequence was carried out in a volume of 50 μl containing 1× HF reaction buffer (Bioline), 2.5 mM MgCl_2_, 200 μM of all four dNTPs (Roche), 0.1 μM each of the forward (TCAGCTATGACCATGATTAC) and reverse (GGCTTAACTATGCGGCATCAGAG) primers (Eurofins MWG Operon, Germany), 25 ng of pSJ1 and 10 nM Tgo-Pol Z1 were used. The amplification of the *lacZα* coding region was achieved in 30 PCR cycles (30 s at 95°C, 35 s at 55°C and 30 s at 70°C). The resulting PCR product was purified by agarose gel electrophoresis (stained with ethidium bromide), recovered using a gel extraction kit (Qiagen) and used to direct a subsequent PCR, where the amplicon served as a ‘megaprimer’ to amplify the entire pSJ1. The second high fidelity PCR was performed in a 50 μl containing 1× HF reaction buffer (Bioline), 2.5 mM MgCl_2_, 200 μM of all four dNTPs (Roche), 1 μg of the *lacZα* ‘megaprimer’, 50 ng of pSJ1 and 20 U/ml Velocity DNA polymerase (Bioline, UK). High Fidelity PCR amplification of pSJ1 using the *lacZα* ‘megaprimer’ was achieved in 20 PCR cycles (30 s at 95°C, 35 s at 55°C and 2.5 min at 70°C). The product of second round of PCR amplification was purified using a PCR clean-up kit (Qiagen, UK) and subjected to Dpn I (Fermentas) restriction endonuclease digestion in order to destroy any parental plasmid DNA. The Dpn I resistant PCR product was transformed into Top 10 *E. coli* (Invitrogen) for blue/white colony analysis. Subsequently, *lacZα* mutants (white colonies) were subject to DNA sequencing across the *lacZα* coding region.

### Fingers domain modelling

Models were derived for the fingers domains of Sce-Pol ζ, Pfu-Pol D473G and Tgo-Pol Z1 using the Protein Homology/AnalogY Recognition Engine V2.0 (‘Phyre2’) (38). These models were generated using the highest homology known structures, selected by the ‘Phyre2’ platform, as templates. The fingers domain of Sce-Pol ζ was predicted with an overall 99.8 confidence index based on 37% identity to Sce-Pol δ (pdb: 3IAY). The fingers domain of Pfu-Pol D473G was predicted with overall 99.8 confidence index based on 83% identity to Pfu-Pol (pdb: 2VWK). The fingers domain of Tgo-Pol Z1 was predicted with overall 99.8 confidence index based on 40% identity to the Sce-Pol δ structure (pdb: 3IAY). The fingers domains of Sce-Pol α (14), Sce-Pol δ (15), Pfu-Pol B (39) and Tgo-Pol B (40) were extracted from previously solved structures. The structures of the fingers domains were aligned using UCSF Chimera software (41).

## RESULTS

### Replication of control primer-templates and primer-templates that terminate in a mismatched base pair

**Figure 3.**
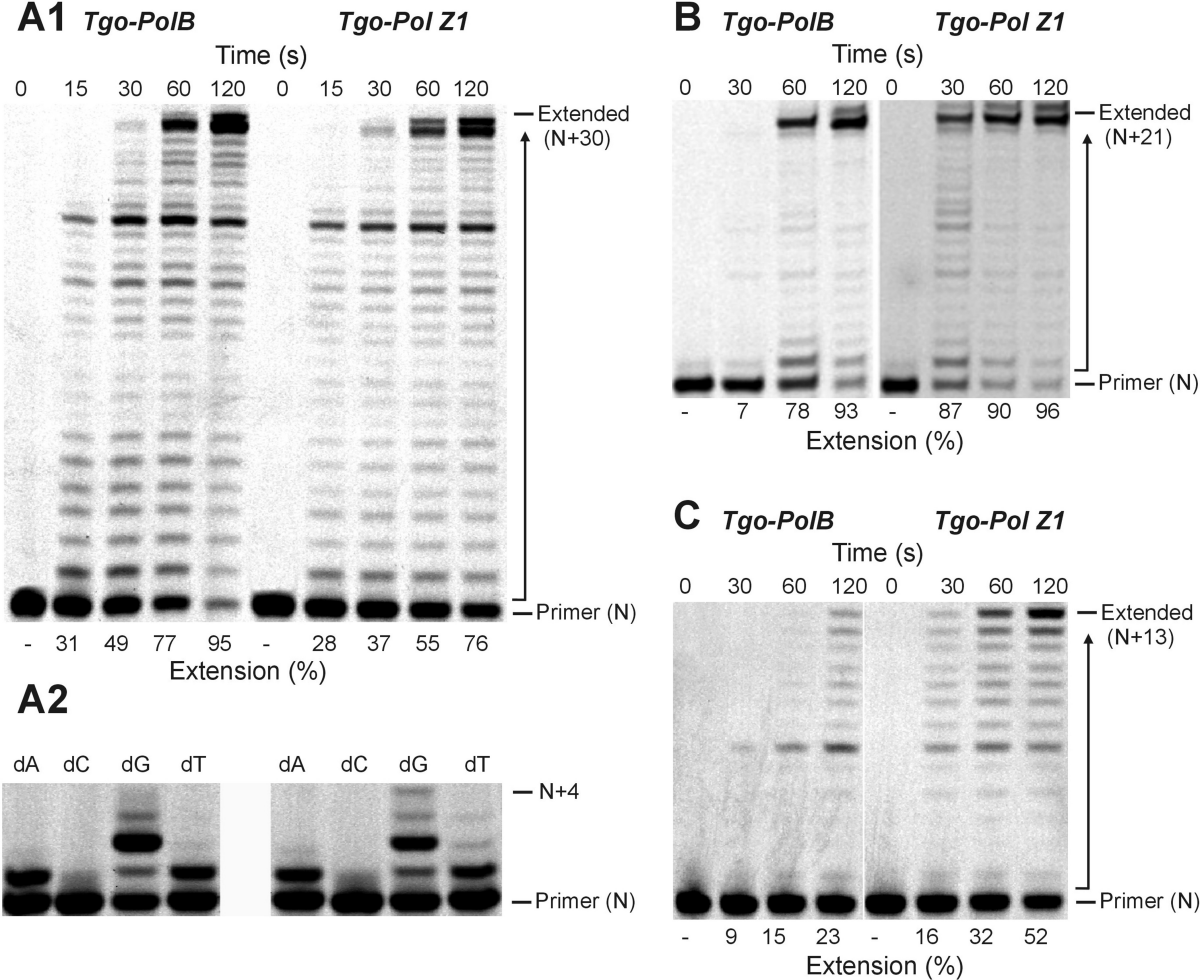
Extension of control DNA and primer-templates that terminate in base mismatches. (**A**) Polymerisation of a control DNA lacking modified bases by Tgo-Pol and Tgo-Pol Z1. The primer-template shown in Figure 2A (X = T, Y = T; running start primer) was used. The main panel (**A1**) shows extension with all four dNTPs added. The smaller panel (**A2**) shows what takes place when only a single dNTP is used (with this primer-template combination, the first and second template bases are both dC; therefore, addition of two dGTPs represents ‘correct’ insertion). (**B**) Extension of a primer-template from a C:T base mismatch (Figure 2B) by Tgo-Pol and Tgo-Pol Z1. (**C**) Extension of a primer-template from a T:T base mismatch (Figure 2C) by Tgo-Pol and Tgo-Pol Z1.

Little difference in activity was observed between Tgo-Pol and Tgo-Pol Z1 (both derivatives are exo^−^) when a standard primer-template was extended, suggesting that altering the fingers loop has little influence on intrinsic activity (Figure 3A). Notably, Tgo-Pol Z1 seems to show slightly lower activity than the parental Tgo-Pol when the primer extension rates were compared. Confirmation came from the near identical performance of the two proteins in PCR reactions (Supplementary Figure S1). Similarly, only minor differences were observed when just a single dNTP was used for extension. Using a template with two cytosine bases immediately ahead of 3′ end of the primer, both polymerases rapidly incorporated, as expected, two dGMPs (Figure 3A). But mis-incorporation of a single dAMP and dTMP (and to a much lesser extent dCMP) was also apparent. Tgo-Pol Z1 may be marginally better at mis-incorporation as shown by the slightly more pronounced bands at +4 in the dG lane and +2 and +3 in the dT lane. With a fully ‘intact’ polymerase, such mismatched bases would normally be excised by the 3′–5′ proof-reading exonuclease activity (42). To further investigate the ability of the polymerases to extend mis-pairs, we used two primer-templates that direct replication from either a mismatched C:T or T:T base pair (Figure 2B and C). Usually, such mistakes occur when a polymerase pairs a template base with an incorrect dNTP during replication. If such errors escape correction by the proof-reading exonuclease activity of the replicating polymerase, subsequent genome copying must commence from the resulting mismatch. Differences between Tgo-Pol and Tgo-Pol Z1 were readily apparent, with Tgo-Pol Z1 extending the mismatched primers noticeably more rapidly than the control with both primer-templates (Figure 3B and C). Thus, the insertion of the Pol ζ fingers domain into Tgo-Pol results in a derivative better able to extend from DNA base mismatches.

### Replication of primer-templates containing a single damaged base

**Figure 4.**
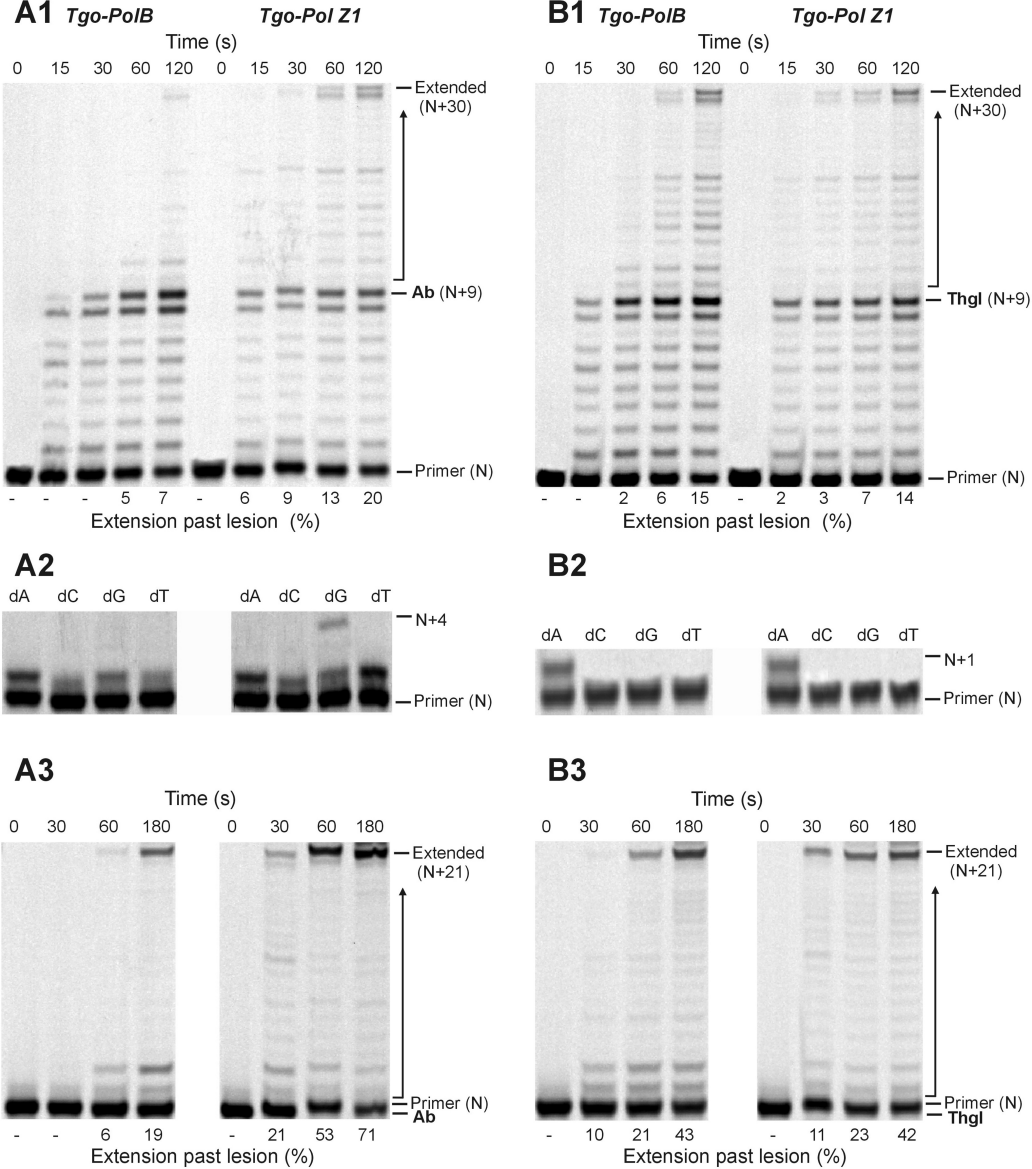
Extension of primer-templates that contain a single damaged base in the template. (**A**) Copying of a template containing an abasic site (Ab) (Figure 2A; X = T, Y = abasic site) by Tgo-Pol and Tgo-Pol Z1. (**A1**) shows the results found with the running start primer and all four dNTPs. (**A2**) uses the primer that initiates polymerisation from just before the abasic site (Figure 2A) and only a single dNTP to determine preferential incorporation opposite the lesion. (**A3**) utilises a primer that has a dA base across from the abasic site (Figure 2A), enabling extension from the dA:abasic site mis-pair to be evaluated. (**B**) Copying of a template containing a thymine glycol (Thgl) (Figure 2A; X = T, Y = thymine glycol) by Tgo-Pol and Tgo-Pol Z1. (**B1**), (**B2**) and (**B3**) are identical to A1, A2 and A3, except a thymine glycol replaces the abasic site.

To investigate the effects of introducing a damaged base into DNA, two types of lesions, an abasic site and a thymine glycol, were selected. Abasic sites can arise spontaneously by depurination/depyrimidation of both standard and damaged bases and are also intermediates in base excision repair (43). Thymine glycols are generated by exposure to ionising and ultraviolet (UV) radiation and reactive oxygen species (44,45). The presence of an abasic site strongly inhibits Tgo-Pol and with a running start primer, noticeable stalled bands at +8 (immediately before the lesion) and +9 (flush with the lesion) (Figure 4A) were produced. This result suggests that the wild-type enzyme has difficulties both inserting a deoxynucleotide across an abasic site and also extending the mismatch produced. However, some read-through is apparent and full-length products are slowly formed. Single deoxynucleotide incorporation, using a primer designed to initiate synthesis immediately prior to the lesion (Figure 2A), shows the wild type prefers to insert purines opposite abasic sites, although pyrimidines can also be incorporated (Figure 4A). Tgo-Pol Z1 is more easily able to replicate through abasic sites; with the running start primer bands at +8 and +9 are apparent, but are less pronounced than for the wild type, and full extension occurs more rapidly. Tgo-Pol Z1 is capable of inserting all four bases opposite the lesion, with little discrimination between purines and pyrimidines (Figure 4A). Tgo-Pol Z1 was better than wild type at continuing synthesis from an abasic site:dA mis-pair, the expected product following initial insertion of dAMP opposite the basic site (Figure 4A). Both enzymes handled the thymine glycol in a near equivalent fashion and this lesion provides much less of a barrier, especially to the wild type, than does the abasic site (Figure 4B). With both the parent and Tgo-Pol Z1, evidence of stalling at +8 and +9 is apparent; nevertheless, full-length product builds up reasonably rapidly. Both polymerases pair the thymine glycol with adenine alone, insertion of the other three bases not taking place, and are able to extend reasonably efficiently from this mis-pair (Figure 4B).

### Replication of primer-templates containing thymine photodimers

**Figure 5.**
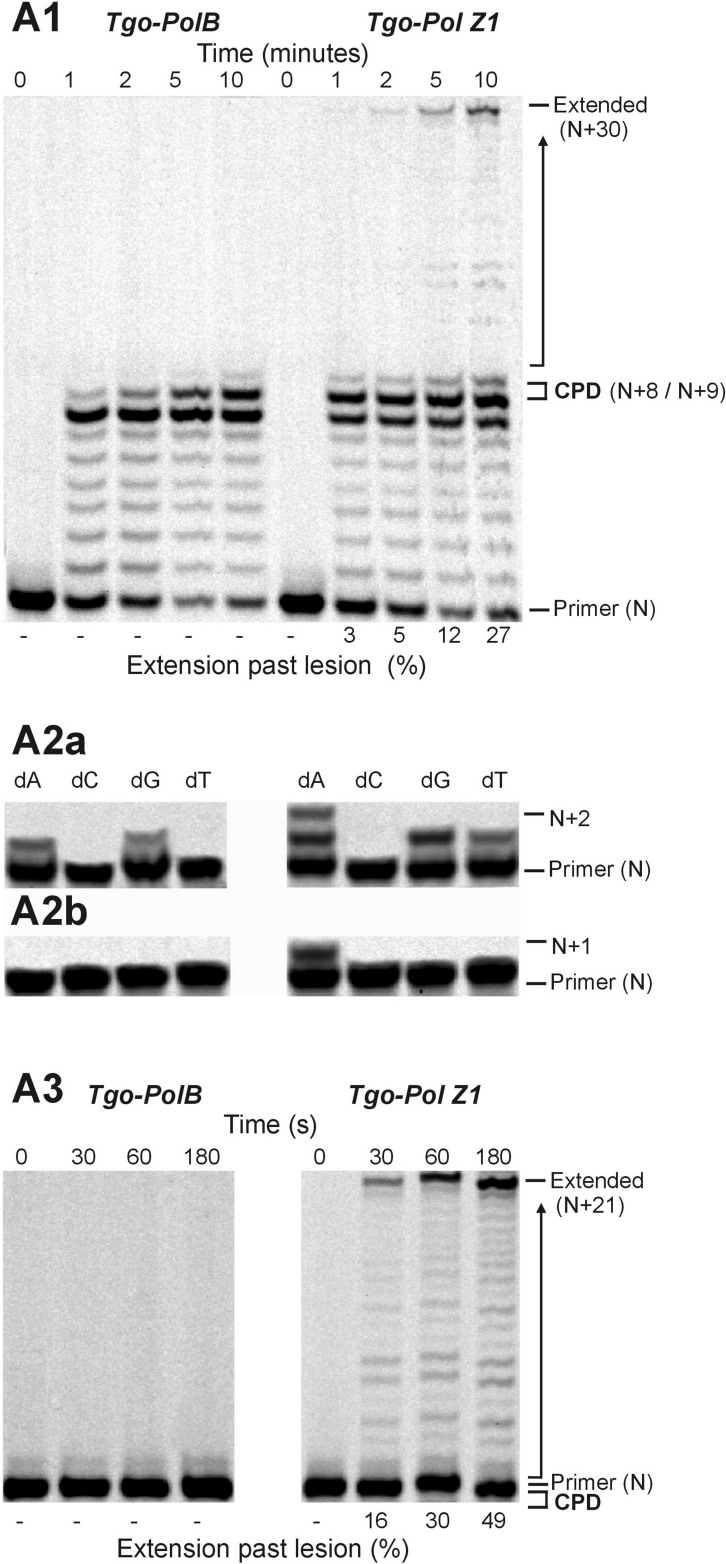
Extension of a primer-template containing a *cis*-syn thymine–thymine cyclobutane pyrimidine dimer (CPD) (Figure 2A; XY = dimer) by Tgo-Pol and Tgo-Pol Z1. (**A1**) Products produced using the running start primer in the presence of all four dNTPs. (**A2**) Products observed using a single dNTP and the primers that initiate synthesis immediately before the lesion (**A2a**) or opposite the first (3′) base of the lesion (**A2b**). (**A3**) Products found with the primer that commences polymerisation at the second (5′) base of the thymine dimer in the presence of all four dNTPs.

Much damage to DNA, e.g. abasic sites and thymine glycol discussed above, requires a polymerase to traverse a single lesion. Exposure of DNA to UV radiation can give rise to the thymine photodimers, the most abundant of which are the cyclobutane pyrimidine dimer (CPD) and the 6–4 photoproduct (6–4pp) (46,47). In these cases, the polymerase has a more demanding task in that it must negotiate two damaged bases. The presence of a thymine–thymine *cis*-syn cyclobutane dimer essentially blocks passage by wild-type Tgo-Pol (Figure 5). With the running start primer, a strong stop was observed at +7 (immediately before the CPD) and a slightly weaker one at +8 (corresponding to copying the first base in the CPD). Further investigation made use of three primers that commence polymerisation from either just before the CPD or at the first or second damaged T within the lesion (Figure 2A). Tgo-Pol was capable of adding a purine opposite the initial lesion base; however, no incorporation at the second position took place and extension from the double mismatch between the templating CPD and two adenines (dAA) of the primer 3′ end was also not possible (Figure 5). The single base incorporations are fully compatible with the running primers results, which indicate pausing just before the CPD and at the first damaged thymine of the lesion. In contrast to the wild type, Tgo-Pol Z1 is able to read through the CPD and full-length product is slowly formed with the running start primer (Figure 5). Using the three primers designed to initiate synthesis at the lesion (Figure 2A) showed that Tgo-Pol Z1 could incorporate dAMP, dGMP or dTMP across the first (3′) damaged thymine and dAMP opposite the second (5′) thymine of the CPD. Additionally, extension from double mismatch between templating CPD lesion and two adenines (dAA) located at 3′ end of the primer was observed in case of Tgo-Pol Z1 (Figure 5).

**Figure 6.**
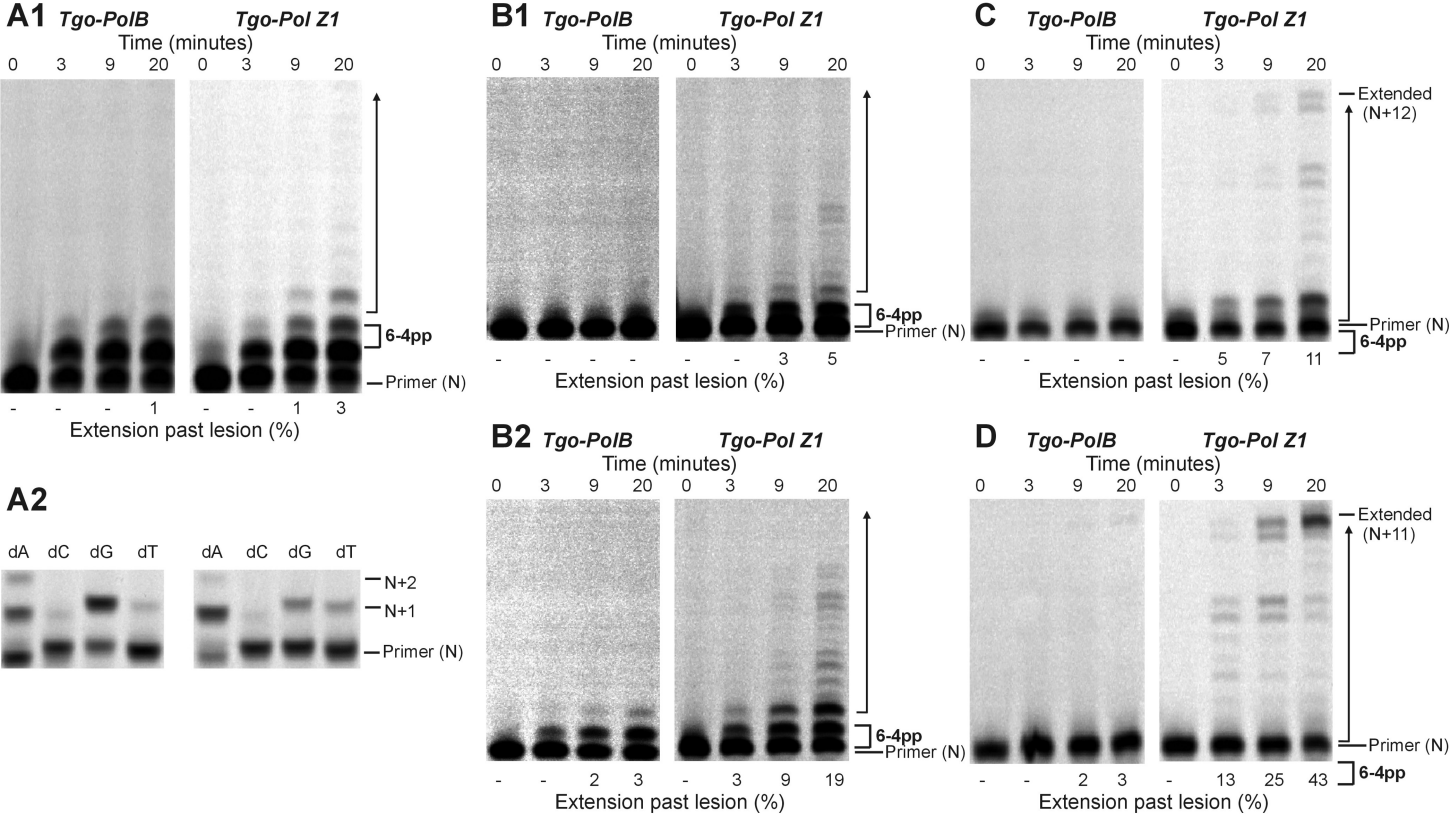
Extension of a primer-template containing a thymine–thymine 6–4 photoproduct dimer (6-4pp) (Figure 2D; ZZ = dimer) by Tgo-Pol and Tgo-Pol Z1. (**A1**) and (**A2**) Products seen using the primer that initiates synthesis immediately before the lesion with all four dNTPs (A1) or a single dNTP (A2). (**B1**) and (**B2**) Products observed with the primer that begins synthesis at the first (3′) base in the 6–4 photoproduct dimer, with the last (3′) base in the primer being either dC (B1) or dA (B2). (**C**) Products produced with the primer that commences polymerisation at the second (5′) base of the lesion. (**D**) Products seen using the primer that begins synthesis at the standard dC base that immediately follows the lesion.

The thymine–thymine 6–4 photoproduct (6–4pp) was a more powerful block to both Tgo-Pol and Tgo-Pol Z1 than the CPD lesion. Due to the difficulties in incorporating the 6–4pp lesion into oligodeoxynucleotides, a short template had to be used and, as a consequence, one primer initiated synthesis from immediately before the lesion (a running start primer would not be long enough to produce a stable duplex) (Figure 2D). With both polymerases, the addition of all four dNTPs resulted in slow addition of one base, followed by a less efficient second incorporation (Figure 6A). With Tgo-Pol Z1, noticeable addition of a third base was also observed. Single dNTP incorporation showed Tgo-Pol preferentially incorporated purines across the first lesion base, although weaker activity with pyrimidines was also apparent. With Tgo-Pol Z1, a similar selectivity was observed, although incorporation of dTMP was more prominent than for the wild-type enzyme. For both enzymes, only dATP led to slow incorporation of two bases to completely cover the 6–4pp lesion (Figure 6A). With a primer that terminated opposite the first (3′) lesion base, further extension was only observed when the deoxynucleotide at the 3′ primer end (the initiation point for synthesis) was dC or dA (Figure 6B). When dC was 3′ terminal base of the primer, Tgo-Pol slowly added a single base; Tgo-Pol Z1 more rapidly incorporated one base and could slowly catalyse further extension. More pronounced activity was observed with dA as the initiating deoxynucleotide. Tgo-Pol introduced one base and, more slowly, a second; Tgo-Pol Z1 incorporated the first base at about the same rate as Tgo-Pol, but was much quicker at adding a second base and also incorporated further deoxynucleotides (Figure 6B). With dG or dT as the 3′ base with these primers, very little incorporation was seen with either polymerase (data not shown). Using a primer that placed two adenines (dAA) across the 6–4pp lesion led to near complete inhibition of Tgo-Pol; in contrast, Tgo-Pol Z1 was able to continue polymerisation (Figure 6C). Finally, very similar results were observed with the longest primer, which covers both the 6–4pp lesion and the first post damage base. Tgo-Pol was unable to extend from this primer whereas Tgo-Pol Z1 showed clear primer extension (Figure 6D).

### Fidelity and mutation spectrum of Tgo-Pol Z1; fidelity of *E. coli* Pol II

Translesion DNA polymerases are typically far more error-prone than the enzymes devoted to replication. Therefore, the fidelity of Tgo-Pol Z1 was determined using a plasmid-based *lacZα* indicator gap-filling assay developed in our laboratory (36,37). These studies used pSJ2, a plasmid that permits the conversion of raw mutation frequencies (ratios of white and blue *E*. *coli* colonies) to an absolute error rate (number of mistakes made per base incorporated) (36). The results are presented in Table 2A and show that fully functional Tgo-Pol (i.e. the wild type with proof-reading exonuclease activity) has an error rate of 2.8 × 10^−6^. A similar value of 1.6 × 10^−6^ has been reported for the closely related Pfu-Pol (37). Disabling the exonuclease activity of Tgo-Pol, with the single mutation D215A (to give the immediate parent of Tgo-Pol Z1), raised to error rate to 1.2 × 10^−5^, about 4-fold higher than the exonuclease-competent enzyme. A comparable drop in fidelity was observed when Pfu-Pol was rendered exonuclease deficient (36). Modification to give Tgo-Pol Z1, which has an altered fingers loop as well lacking exonuclease activity, further elevated the mutation rate to 1.9 × 10^−5^. Thus, the fidelity of Tgo-Pol Z1 is ∼1.6 times lower than its immediate precursor. Previously, we determined that Pfu-Pol exo^−^ (D215A/E143A), with an additional single mutation (D473G) in its fingers loop, has an error rate of 1.8 × 10^−5^, almost identical to that observed with Tgo-Pol Z1 (36).

**Table 2A.** Error rates of Tgo-Pol (exo^−^) and Tgo-Pol Z1 (exo^−^) determined using pSJ2^a^ or pSJ3^a^ (a plasmid-based fidelity assay employing the *lacZα* indicator gene)

Protein	Total number of colonies^b^	Number of white (mutant) colonies	Corrected mutation frequency^c^	Error rate^d^
Tgo-Pol (exo^+^)	29 271	20	5.7 × 10^−4^	2.8 × 10^−6^
Tgo-Pol (exo^−^: D215A)	62 656	155	2.4 × 10^−3^	1.2 × 10^−5^
Z1-Tgo-Pol (exo^−^: D215A)	64 657	266	4.0 × 10^−3^	1.9 × 10^−5^
Pfu-Pol (exo^−^: E143A/D215A)(D473G)^e^	78 431	296	3.7 × 10^−3^	1.8 × 10^−5^
Eco-Pol II (exo^+^)	59 235	28	4.4 × 10^−4^	3.0 × 10^−6^
Eco-Pol II (exo^−^: D335N)	41 903	128	3.0 × 10^−3^	2.1 × 10^−5^

^a^pSJ2 was used with the archaeal enzymes, pSJ3 with *E*. *coli* DNA polymerase II.

^b^The fidelity of each protein was determined in three separate experiments, each of which involved scoring *E. coli* colonies on five separate plates. The aggregated numbers are given.

^c^Corrected mutation frequency equals: ({number of white colonies/total number of colonies} – background mutation rate). Background mutation rates of 1.1 × 10^−4^ and 3.1 × 10^−5^ were used for gapped pSJ2 and pSJ3, respectively (36).

^d^Error rate is the number of mistakes made per base incorporated. The corrected mutation frequency was converted to the error rate as previously described.

^e^Data taken from an earlier publication (36).

To further investigate the low fidelity of Tgo-Pol Z1, the enzyme has been used to amplify the *lacZα* sequence in pSJ1 (37). Using this PCR-based approach had significant advantage over the *lacZα* gap-filling assay. It allowed efficient and straightforward investigation of the mutation type introduced into the amplicon. Simultaneously, the performance of Tgo-Pol Z1 in error-prone PCR was assessed. The mutagenic nature of Tgo-Pol Z1 results in a highly error-prone PCR and about 5.5% of *E*. *coli* colonies, which are ultimately generated from the PCR product, have an inactivated *lacZα* gene (white phenotype). For comparison, the parental Tgo-Pol exo^−^ yields about 1.5% white colonies. Sequencing of 17 randomly selected mutant colonies revealed a mutagenic load of approximately 19 alterations per kb of amplified PCR product. In total, 108 changes to the parental sequence were observed (Supplementary Figure S2) and are summarised in Table 2B. During the PCR, Tgo-Pol Z1 shows a high propensity for mutating G:C base pairs, which are altered about nine times more frequently than A:T pairs. In contrast, transversion mutations are only slightly favoured over transitions. The Pfu-Pol exo^−^ D473G variant gives rise to a lower mutagenic load (estimated to 7.1 mutations per kb) but shows an almost unbiased mutation spectrum, suggesting better suitability in error-prone PCR applications (33).

**Table 2B.** Individual changes introduced into the *lacZα* indicator gene of pSJ1 by Tgo-Pol Z1 (exo^−^)

Mutation type	Number	Frequency (%)			
A→T/T→A	8	7.4
A→C/T→G	0	0
A→G/T→C	2	1.9
G→A/C→T	37	34.3
G→C/C→G	37	34.3
G→T/C→A	12	11.1
Insertions	5	4.6
Deletions	7	6.5
Total	108	100
A→N/T→N	10	10.4
G→N/C→N	86	89.6
Transitions	39	40.6
Transversions	57	59.4

A total of 108 white colonies were completely sequenced in order to reveal the actual change in the DNA responsible for the white phenotype.

Previous studies on the fidelity of *E. coli* DNA polymerase II had been conducted using primer-template-based assays (48). The collected data suggested that Eco-Pol II is about 2–6 times more error-prone than main *E. coli* replicase (Eco-Pol III). Interestingly, nucleotide mis-incorporation frequencies for Eco-Pol II were found to be 3–5-fold higher in AT- compared with GC-rich DNA (48). As accuracy of Eco-Pol II has not previously been determined using *lacZα* gap-filling assay, we were encouraged to study fidelity of this polymerase using the pSJ3 plasmid fidelity assay; the results are given in Table 2A. Additionally, these data are also important for full interpretation of our results in the Discussion section where fidelities of archaeal family-B DNA polymerases and its error-prone/translesion derivatives will be compared with Eco-Pol II. The observed error rate of Eco-Pol II was 3.0 × 10^−6^, almost identical to wild-type Tgo-Pol. Disabling the proof-reading exonuclease activity of Eco-Pol II reduced fidelity by about an order of magnitude, again similar to values observed for Tgo-Pol exo^−^. The archaeal family-B DNA polymerases, like Tgo-Pol, are normally considered high fidelity enzymes and on this basis, Eco-Pol II also appears to be very accurate.

## DISCUSSION

The segregation of replicative and translesion DNA polymerases into distinct sub-groups is not absolute, as both categories are found within the B-lineage. How is a single family of enzymes, with considerable amino acid similarity and structural homology, able to accommodate the different properties of the replicative and translesion polymerases? High-resolution crystallographic structures of *E. coli* DNA polymerase II (Eco-Pol II), and comparison with the family-B replicative polymerase from the T4 bacteriophage (gp43), have, in this instance at least, provided an answer (27). Eco-Pol II is better able to read through abasic sites and extend from mismatches than gp43, a gain of function that has its origin in the presence of 20 additional amino acids in the N-terminal domain. The insertion modulates partitioning DNA substrate between polymerase active site and 3′–5′ exonuclease proof-reading domain and ultimately leads to formation of small cavities near the template strand, capable to accommodate aberrant bases and allow subtle re-adjustments of the primer-template, favouring translesion synthesis. The N-terminal expansion also impedes movement of the primer to the proof-reading exonuclease site, hindering removal of any cognate bases inserted opposite damage, which would reduce translesion synthesis (27). Despite these features, Eco-Pol II handles normal DNA with reasonable efficiency and has high fidelity, comparable to that of Pfu-Pol and Tgo-Pol (Table 2A), properties important in its role in replication re-start in an error-free manner (26,49).

The second translesion family-B polymerase, Sce-Pol ζ, is inefficient at running start replication through damaged bases including the *cis*-syn cyclobutane and (6–4) photo dimers of thymine, thymine glycol, abasic sites, 8-oxo-dG and O6-methyl-dG, although with some of these lesions full-length product is slowly formed (50–56). Rather, Sce-Pol ζ is adapted to continue DNA synthesis from a distorted primer-template terminus that may arise from (i) a mistake by a replicative polymerase giving a mismatched base pair; (ii) a family-Y translesion polymerase inserting cognate base(s) across the damaged site. The action of two polymerases may be necessary for efficient read-through of aberrant bases, initial copying by a Y-family polymerase and subsequent extension by Sce-Pol ζ (31). Unfortunately, there is no crystal structure of Sce-Pol ζ available; therefore, a molecular explanation for its propensity to distorted primer-templates remains unknown. It has been proposed that factors determined to be important with Eco-Pol II may play a role; Sce-Pol ζ has the N-terminal insertion, perhaps leading to cavity formation near the 3′ end of primer and tolerance of damaged templating nucleobases (27). Sce-Pol ζ may conceivably also make use of features found in family-Y polymerases. Several structures (see reviews 9–10,31) have shown a smaller thumb and fingers domain plus an additional feature of ∼100 amino acids, the little finger domain. As a consequence, the active site of Pol ζ might be solvent exposed with an increased surface to readily accommodate damaged bases and mismatches.

We observed that shortening of the loop in the fingers domain of archaeal family-B polymerases (Figure 1) resulted in translesion synthesis-like properties. Single amino acid substitutions reduced fidelity (33) and more drastic loop transplantation of the region found in Sce-Pol ζ, yielding Tgo-Pol Z1, resulted in reverse transcriptase activity (34). If the loop swap had converted the high fidelity archaeal polymerase to a Pol ζ-like enzyme, the behaviour of Tgo-Pol Z1 should resemble Sce-Pol ζ. Gratifyingly, Tgo-Pol Z1 is better at initiating polymerisation from mismatches than Tgo-Pol (exo^−^) from which it derives (Figure 3), behaviour characteristic of Sce-Pol ζ (50,56). Similarly, Tgo-Pol Z1 is competent at initiating from primers that pair dA with an abasic site (Figure 4) and two dAs with the CPD and the 6–4pp thymine dimers (Figures 5 and 6), again properties reminiscent of Sce-Pol ζ (50,52). Tgo-Pol Z1 also elongates a primer that places a standard base immediately after the 6–4pp dimer (Figure 6). Here the primer-template junction is likely to remain considerably distorted and require translesion-type properties for efficient extension. Sce-Pol ζ is capable of reading through thymine glycols in an error-free manner, inserting adenine opposite the lesion and continuing extension from the mismatch (53). Tgo-Pol Z1 demonstrates identical properties but, in this instance, the behaviour cannot be assigned to loop insertion as parental Tgo-Pol (exo^−^) has identical properties (Figure 4). Yeast Pol ζ has lower fidelity (error rate 120 × 10^−5^) than replicative B-family polymerases (error rates of yeast Pol α, δ and ϵ are 12.7, 7.0 and 31.6 × 10^−5^, respectively) (11); likewise, Tgo-Pol Z1 (error rate 1.9 × 10^−5^) is less accurate than the high fidelity Tgo-Pol (exo^+^) from which it is derived (error rate 2.8 × 10^−6^) (Table 2). However, the properties of Tgo-Pol Z1 and Sce-Pol ζ diverge in several aspects; most importantly, yeast polymerase ζ is inadequate at direct insertions opposite damaged bases, and is, therefore, poor at running start replication through aberrant DNA (31,50–55). In contrast, Tgo-Pol Z1 is relatively efficient at reading through abasic sites and the CPD dimers (Figures 4 and 5). However, the 6–4 photoproduct comprehensively blocks Tgo-Pol Z1 (Figure 6), as it does Sce-Pol ζ (51), unless the damage is already copied. Overall, lengthening the fingers loop of Tgo-Pol produces an enzyme with some, but not all, of the characteristics of Sce-Pol ζ. Tgo-Pol Z1 is able to initiate from mismatches and lesions already ‘covered’ with cognate bases, the defining trait of Sce-Pol ζ. However, Tgo-Pol Z1 is capable of carrying running start replication through damage, a function Sce-Pol ζ mostly lacks. Perhaps, the observed discrepancy in bypass of CPD between Sce-Pol ζ and Tgo-Pol Z1 might be explained by potential differences in processivity between the compared enzymes.

The well-documented function of the fingers domain allows rationalisation of the acquired ability of Tgo-Pol Z1 to tackle damaged bases. In all polymerases, the fingers domain consists of two anti-parallel α-helices and is responsible for accurate matching of the incoming dNTP with template bases to produce Watson–Crick base pairs. Conserved amino acids (shown in red in Figure 1 and located on the same side of both helices) interact with the dNTP and trigger a large motion of the entire fingers domain to produce a catalytically active ‘closed’ polymerase/DNA/dNTP ternary complex (2,4,57). In high fidelity polymerases, the conformational change, and hence extension, requires correct partnering of dNTP and templating base, a feature that ensures accuracy (58,59). Altering the loop connecting the helices of the fingers domain probably influences the relative dispositions of the dNTP-contacting amino acids, maybe by perturbing the hydrogen bonds formed between the loop aspartate and amino acids in the subsequent α-helix (33). In turn, the resulting more flexible fingers domain may permit the conformational change even when correct Watson–Crick base pairs are not produced. A polymerase with these features would be expected to incorporate cognate bases across DNA lesions and extend from the resulting distorted primer-template. Such an enzyme would also have the propensity to generate mismatches during normal replication, resulting in reduced fidelity.

Within archaeal DNA family-B DNA polymerases, the fingers domain has short and rigid loop region (three amino acid) connecting N- and O- α-helices. The observed characteristic of the loop was associated with high fidelity DNA synthesis of archaeal family-B DNA polymerases. Previously, we have reported that either D473G point mutation or insertion of a longer sequence within the loop region of Pfu-Pol and Tgo-Pol, respectively, shifts properties of these archaeal replicases towards error prone/translesion synthesis characteristics. However, it is difficult to fully generalise loop length and hydrogen bonding potential with replicative/translesion features (Figure 1, Table 1). Some accurate enzymes (Sce-Pols α and δ, Pfu- and Tgo-Pol) have a near identical short loop with a conserved aspartic acid that makes hydrogen bonds to the following α-helix. Models, which need to be interpreted with restraint, suggest many error-prone/translesion polymerases (Sce-Pol ζ, Pfu-Pol (D473G) and Tgo-Pol Z1) have a longer loop and, in some cases, a paucity of hydrogen bonds. A structure of Sce-Pol ζ and perhaps the ‘reverse’ loop swap, incorporating a short sequence into this polymerase, would be highly instructive. Nonetheless, two family-B polymerases, *E*. *coli* Pol II and yeast Pol ϵ, deviate from the simple paradigm. Eco-Pol II is not a replicative polymerase, rather it tolerates damaged bases and is used for replication re-start (26,27). The protein has a three amino acid loop and, although the aspartate is replaced by asparagine, forms hydrogen bonds comparable to the replicative polymerases. Unusually, Eco-Pol II possesses 3′–5′ proof-reading activity and exhibits similar fidelity to Tgo-Pol (Table 2), a replicative-like property. Sce-Pol ϵ has been established as the leading strand replicase and displays high fidelity (11,13), despite the presence of a long loop and a single hydrogen bond to the downstream α-helix. Unexpectedly, Sce-Pol ϵ reads through 7,8-dihydro-8-oxoguanine, O6-methylguanine and abasic sites, features commonly associated with translesion polymerases (60,61). Some polymerases may require a blend of replicative and translesion features for effective *in vivo* performance, explaining the atypical loops of Eco-Pol II and Sce-Pol ϵ. Eco-Pol II seems to be required to carry out a degree of accurate synthesis following replication re-start in *E*. *coli*, prior to handover to the main bacterial replicase (Eco-Pol III). Stalling at the leading strand necessitates assembly of a re-start apparatus and read-through of damaged bases by Sce-Pol ϵ, especially if error-free, may be advantageous in certain circumstances. The lagging strand, copied by Sce-Pol δ, is less problematic as re-start using the next Okazaki fragment may be automatic, allowing replication to continue and subsequent lesion repair (62).

Although fingers domain loop extension gives a translesion-type polymerase with the archaeal enzymes, such a change on its own may not generate suitable *in vivo* features. Whether composed of one or two polymerases (31), an ideal translesion system should copy the damage and add a few cognate bases to restore the primer-template to standard B-DNA geometry. At this point, the translesion enzymes should disengage to allow high fidelity synthesis by replicative polymerases. Clearly, Tgo-Pol Z1 shows new unique blend of enzymatic features characteristic for both translesion and replicative polymerases. Tgo-Pol Z1 handles damaged DNA bases and it is also very efficient at copying normal DNA. Notably, Tgo-Pol Z1 is also significantly more error-prone than the parental Tgo-Pol. The above observation proves that manipulation of the loop is a fruitful strategy for generating highly active thermostable polymerases with novel desired features. Pfu-Pol D473G is useful in error-prone PCR (33) and has been recently used to generate a library of *E*. *coli* FtsZ mutants (63). Tgo-Pol Z1 has reverse transcriptase activity, potentially applicable in RT-PCR (34), and it can efficiently read through DNA damage. The translesion properties of Tgo-Pol Z1 might find application in the amplification from partially degraded DNA. However, given the low fidelity of Tgo-Pol Z1, further experimental evaluation is need before use of this polymerase in the specialised PCR application.

Interestingly, recent efforts in re-engineering of Tgo-Pol properties using compartmentalised self-replication (CSR) resulted in a novel variant of this polymerase possessing translesion DNA polymerase and DNA template dependent RNA polymerase activities (64). In this study, Cozen and colleagues demonstrated that E664 residue (located in thumb domain) acts as a steric gate, which is involved in recognition of the DNA substrate by Tgo-Pol. It would be logical to introduce the above point mutation into Tgo-Pol Z1 to further relax substrate recognition of this polymerase.

## SUPPLEMENTARY DATA

Supplementary Data are available at NAR Online.

SUPPLEMENTARY DATA
