# Supplementary material for: An archaeal family-B DNA polymerase variant able to replicate past DNA damage: occurrence of replicative and translesion synthesis polymerases within the B family
Source: Nucleic Acids Res. 2014 Jul 24;42(15):9949–63. doi: 10.1093/nar/gku683 (PMC4150786; doi:10.1093/nar/gku683)
Supplement: SUPPLEMENTARY DATA [file supp_42_15_9949__index.html]

An archaeal family-B DNA polymerase variant able to replicate past DNA damage: occurrence of replicative and translesion synthesis polymerases within the B family — An archaeal family-B DNA polymerase variant able to replicate past DNA damage: occurrence of replicative and translesion synthesis polymerases within the B family — SUPPLEMENTARY DATA 

# An archaeal family-B DNA polymerase variant able to replicate past DNA damage: occurrence of replicative and translesion synthesis polymerases within the B family

## SUPPLEMENTARY DATA

**Files in this Data Supplement:**

- SUPPLEMENTARY DATA
