# Supplementary material for: An archaeal family-B DNA polymerase variant able to replicate past DNA damage: occurrence of replicative and translesion synthesis polymerases within the B family
Source: Nucleic Acids Res. 2014 Jul 24;42(15):9949–63. doi: 10.1093/nar/gku683 (PMC4150786; doi:10.1093/nar/gku683)
Supplement: SUPPLEMENTARY DATA [file supp_gku683_nar-01167-h-2014-File008.pdf]

SUPPLEMENTARY DATA: FIGURE S1

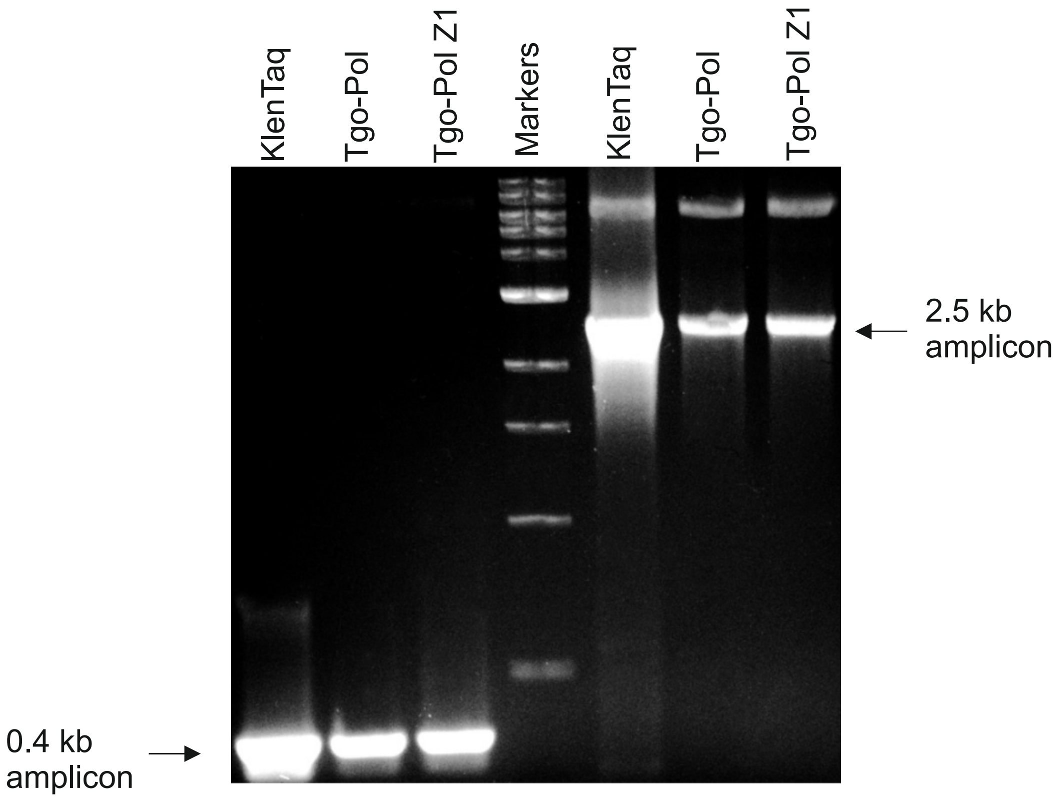

Amplification of 0.4 and 2.5 kb targets within *Archaeoglobus fulgidus* genomic DNA was carried out with KlenTaq, Tgo-Pol (exo^{-}) and Tgo-Pol Z1 as described in the main text. Analysis was by 1% agarose gel electrophoresis stained with ethidium bromide.

SUPPLEMENTARY DATA: FIGURE S2

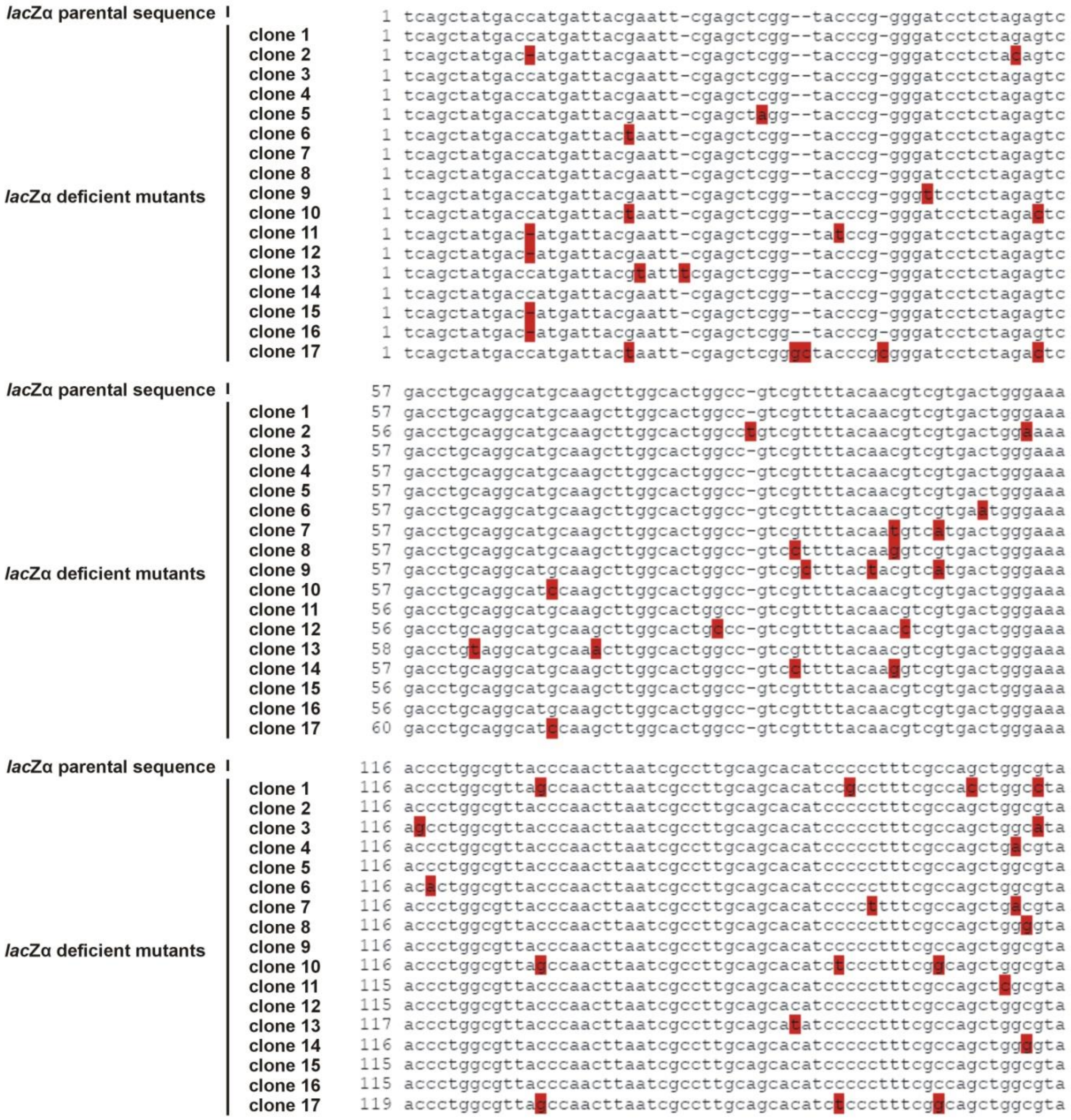

SUPPLEMENTARY DATA: FIGURE S2 (CONTINUED)

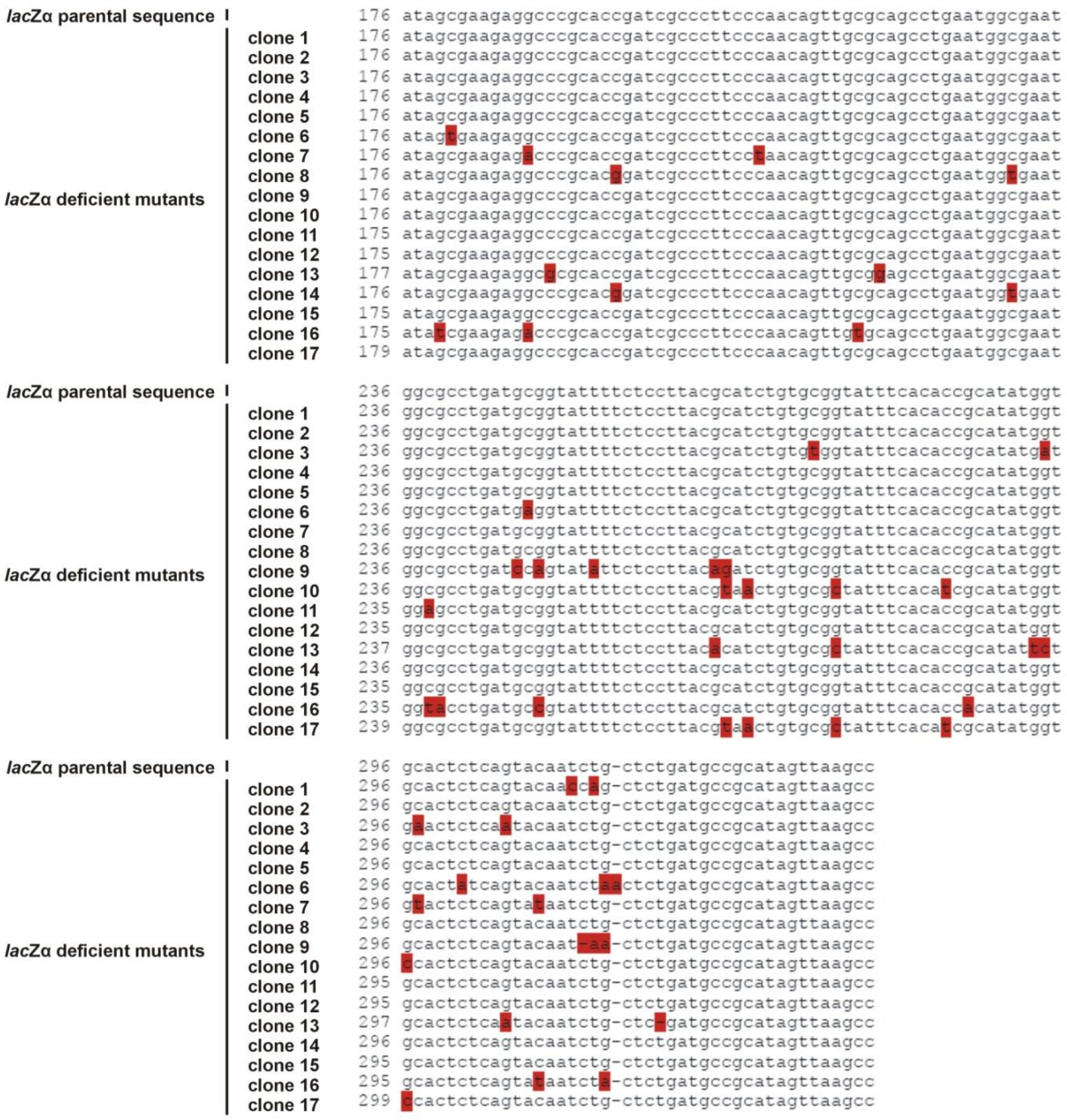

Alignment of the *lacZ*α parental gene in pSJ1 with sequences obtained from seventeen mutant (white) colonies during pCR with Tgo-Pol Z1 (see main text for more details). Alterations are indicated in red.
